# Characterising and differentiating cognitive and motor speed in older adults: structural equation modelling on a UK longitudinal birth cohort

**DOI:** 10.1136/bmjopen-2024-083968

**Published:** 2024-08-19

**Authors:** Indra Bundil, Sabina Baltruschat, Jiaxiang Zhang

**Affiliations:** 1 School of Psychology, Cardiff University, Cardiff, UK; 2 Department of Computer Science, Swansea University, Swansea, UK

**Keywords:** aging, behavior, psychometrics, retrospective studies, task performance

## Abstract

**Abstract:**

**Objectives:**

Information processing speed (IPS) has been proposed to be a key component in healthy ageing and cognitive functioning. Yet, current studies lack a consistent definition and specific influential characteristics. This study aimed to investigate IPS as a multifaceted concept by differentiating cognitive and motor IPS.

**Design, setting and participants:**

A retrospective data analysis using data from the Medical Research Council National Survey of Health and Development (a population-based cohort of UK adults born in 1946) at childhood (ages 8, 11 and 15) and adulthood (ages 60–64 and 68–70). Using structural equation modelling, we constructed two models of IPS with 2124 and 1776 participants, respectively.

**Outcome measures:**

Measures of interest included IPS (ie, letter cancellation, simple and choice reaction time), intelligence (ie, childhood intelligence and National Adult Reading Test), verbal memory, socioeconomic status (SES) and cognitive functions measured by the Addenbrooke’s Cognitive Examination III, as well as a variety of health indexes.

**Results:**

We found distinct predictors for cognitive and motor IPS and how they relate to other cognitive functions in old age. In our first model, SES and antipsychotic medication usage emerged as significant predictors for cognitive IPS, intelligence and smoking as predictors for motor IPS while both share sex, memory and antiepileptic medication usage as common predictors. Notably, all differences between both IPS types ran in the same direction except for sex differences, with women performing better than men in cognitive IPS and vice versa in motor IPS. The second model showed that both IPS measures, as well as intelligence, memory, antipsychotic and sedative medication usage, explain cognitive functions later in life.

**Conclusion:**

Taken together, these results shed further light on IPS as a whole by showing there are distinct types and that these measures directly relate to other cognitive functions.

**STRENGTHS AND LIMITATIONS OF THIS STUDY**

- A large longitudinal cohort data set with different measurements of information processing speed (IPS) that are widely used.
- IPS is not only related to variables measured at the same time but also to childhood and premorbid intelligence and cognitive functions in later life.
- Limitations of the cohort dataset include different response rates between waves, thus some variables were not available for all individuals at certain time points, and IPS scores were derived from a small number of trials.
- The study involved self-reported measures, which might have increased the proportion of misclassification.

## Introduction

 Information processing speed (IPS) is conventionally defined as the speed with which individuals sense, perceive, understand and respond to information^1^ and is shown to play a crucial role for cognitive capacity and healthy ageing.^2^,^8^ Furthermore, IPS, assessed via Inspection Time (IT), the Wechsler Adult Intelligence Scale (WAIS) III Symbol Search and Digit-Symbol tests, Simple Reaction Time (SRT) and Choice Reaction Time (CRT), is significantly pairwise correlated with Logical Memory (Wechsler Memory Scale), Verbal Fluency, National Adult Reading Test (NART) and the Wechsler Test of Adult Reading and Letter Number Sequencing (WAIS-III).^9^ These associations emphasise the importance of IPS for higher-order cognitive functioning. Moreover, research on longitudinal data confirmed a decline in IPS during ageing,^10^ suggesting that IPS might serve as a buffer for age-related cognitive decline. Previous research within the Lothian Birth Cohort (LBC) 1936 has demonstrated that IPS in 70-year-olds, measured through IT, SRT and CRT, serves as an indicator for intelligence, spatial and verbal abilities.^9^ However, other studies produced slightly different results: while IPS, as assessed via IT, SRT and CRT, was associated with general cognitive abilities, only SRT and CRT, but not IT, related to childhood intelligence.^5^ This discrepancy highlights the need for a comprehensive approach to investigate IPS and its associations with cognitive and demographic variables.

Education is another demographic variable, with a complex array of previous findings regarding IPS. On one hand, substantial evidence links cognitive functions, including IPS, to educational attainment. For instance, the WAIS-IV standardisation study reported a mean processing speed index of 86 for individuals with less than 8 years of education, compared with 106 for those with more than 18 years.^11^ Zhang *et al*^12^ also found significant associations between IPS (measured by the Digit Symbol Substitution Test (DSST)) and education. On the other hand, a growth curve modelling study showed that the rate of cognitive decline, including IPS, is not associated with educational level.^13^

Heterogeneous findings may be due to the focus on a few selected variables for investigation, without accounting for their covariance with additional variables. For instance, Ritchie *et al*^14^ found that the correlation between IPS (measured via SRT, CRT and IT) and education became non-significant after controlling for childhood IQ, measured prior to differential education at age 11. These findings suggest that while later life IPS is linked to education, childhood IQ (likely indicative of subsequent educational attainment) emerges as the primary determinant. Additionally, this raises the question of whether IPS is independently influenced by education or if observed associations are due to shared variance with intelligence.

Regarding sex differences in IPS, some studies showed that females are faster, for example, in tests involving digits and rapid naming tasks^8 15 16^ while others reported males to perform better, for example, in the Finger Tapping Test (FTT)^17^ and Reaction Time Tasks (RTTs).^18 19^ This variability, again, underscores the need for a statistical model integrating various demographic and cognitive variables to determine their individual and collective influence on IPS.

Discrepancies in IPS findings may also stem from different measurement approaches. IPS is commonly assessed using two main methodologies. First, IPS is often measured with classic neuropsychological cognitive batteries such as the WAIS or Letter Cancellation Test (LCT). In these tasks, the crucial outcome variable is performance (eg, accuracy). In other words, time is constant, that is, participants have X minutes to work on a task and the accuracy highly differs between individuals as a result. The second approach often found in studies is assessing response times, that is, classical RTTs with varying number of choices and thus difficulty level. Performance is not as important for these tests because, in most cases, there is a ceiling effect.^20^ Additionally, tasks profoundly differ in the extent to which they capture cognitive and motor processes. Despite their distinct characteristics, there has been limited research systematically comparing these IPS constructs, leading to gaps in our understanding of their differential contributions to cognitive functioning and demographic factors.

Together, the inconsistencies in findings underscore the necessity of not only examining pairwise correlations between variables but also considering their covariances within a statistical model. Consequently, the primary aim of this study is to employ structural equation modelling (SEM) to delineate the relationships between specific variables, accounting for their covariances with correlated cognitive and demographic factors. To achieve this, we set out with two main objectives: (1) to model individual differences in IPS measures at ages 60–64 (LCT, SRT/CRT) with variables known to be associated with IPS (model 1) and (2) to investigate the longitudinal association of IPS with cognitive decline at ages 68–70 while controlling for health-related, demographic and cognitive variables (model 2).

We use data from the Medical Research Council National Survey of Health and Development (MRC NSHD) cohort, which includes measurements similar to those of the LBC 1936. By modelling separate variables to capture cognitive and motor IPS, we aim to better understand their distinct and overlapping components, addressing a gap in the literature where IPS is often treated as a single construct.

## Method

### Cohort data

The MRC NSHD cohort study is a population-based cohort of originally 5362 British-born participants in March 1946. Data were collected in multiple waves including several ages in childhood (ages 8, 11 and 15) and adulthood (eg, ages 43, 60–64 and 68–70). Data primarily used in this study are from the waves of 2006–2010 (ages 60–64, *M*=63.37, *SD*=1.10) and 2014–16 (ages 68–70, *M*=69.50, *SD*=0.23). The time of collection of each individual variable is listed in the ‘Variables section’. To model different types of IPS, we required complete datasets of all IPS variables measured at ages 60–64 (SRT, CRT and LCT), resulting in 2124 (1114 females) cohort members.

The procedure and original protocol have been reported elsewhere^21^,^23^ and the data of the MRC NSHD cohort are available to researchers on request via a standard application procedure. Further details can be found at http://www.nshd.mrc.ac.uk/data.

### Patient and public involvement

Patients and/or the public were not involved in the design, or conduct, or reporting, or dissemination plans of this research.

### Variables

The variables used in the current study are described below; more details on each of these measures can be found in Moulton *et al*.^21^ Furthermore, we provide a comprehensive evaluation of the psychometric properties of the predominant measures at the end of this section.

1. Information processing speed

1.1 Letter Cancellation Test (LCT)

**Figure 1:**
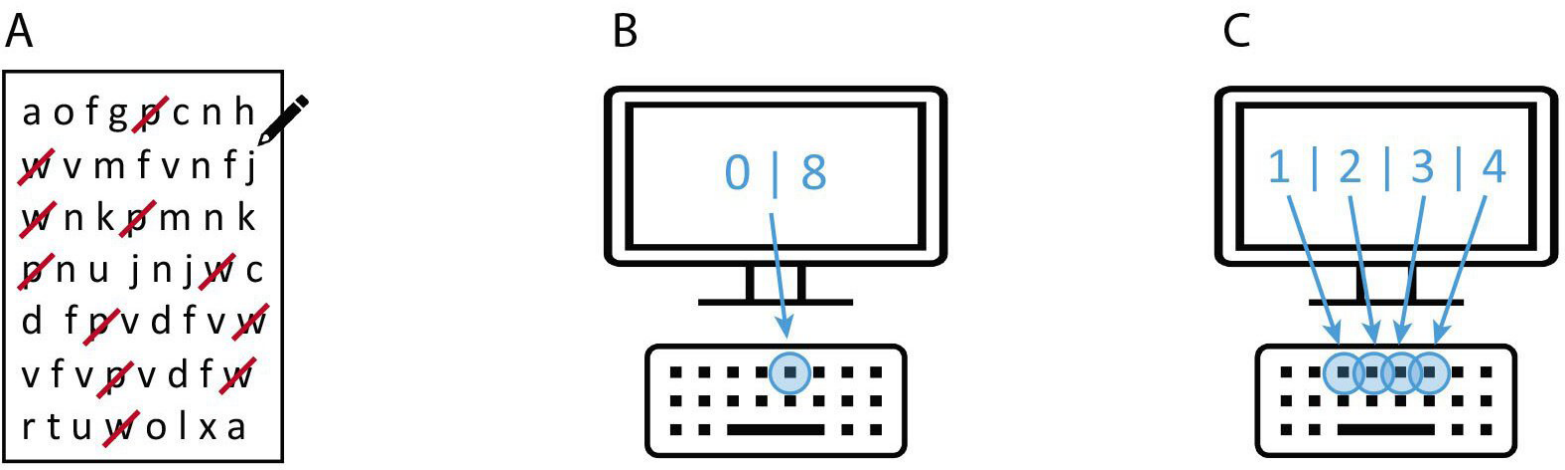
IPS tasks. (**A**) LCT. Participants needed to detect and cross out the letters ‘p’ and ‘w’ on a sheet of paper among many different letters. (**B**) SRT. Participants needed to press the same key when the digit ‘0’ or ‘8’ appeared on the screen, (**C**) CRT. Participants needed to press the respective keys for digits ‘1’, ‘2’, ‘3’ and ‘4’ appearing on the screen. CRT, choice reaction time; IPS, information processing speed; SRT, simple reaction time.

- The task was to detect and cross out the letters ‘p’ and ‘w’ on a sheet of paper among many different letters for a duration of 1 min (see figure 1A). Performance was quantified as the number of letters searched. The hit rate (correct letters found) did not need to be considered as a separate measure, as most participants made no errors. This task was assessed at ages 60–64.

1.2 Simple Reaction Time (SRT)

- A computerised version of the task was used in which participants had to press a specific key as quickly as possible (with only one finger) when the digits ‘0’ or ‘8’ were displayed on the screen (see figure 1B). After a practice of 8 trials, the main test of 20 trials was used to calculate the mean reaction times (measured in milliseconds). This task was assessed at ages 60–64.

1.3 Choice Reaction Time (CRT)

- In the CRT task, one digit from 1 to 4 appeared on screen, and the corresponding key had to be pressed with any finger (see figure 1C). Again, a practice phase of 8 trials preceded the main test of 40 trials. Mean reaction times were calculated (measured in milliseconds). This task was assessed at ages 60-64.

1. Memory

- After being presented a list of 15 words, participants were asked to write down all the words they remembered for 1 min. This process was repeated three times. The total score is the sum of all correctly remembered words. This task was assessed at ages 60–64.

1. Intelligence

- For childhood intelligence, the standardised summary of all intelligence measures at age 8 was used. If the intelligence scores at age 8 were missing, the measurements from age 11 or 15 were used (in 91 participants). Childhood intelligence measures of all ages differed according to age abilities, but all comprised a cognitive battery entailing picture intelligence (eg, finding the odd one out or completing a pattern), reading/word comprehension (eg, completing a sentence with a word out of 5 options), word reading and vocabulary, and arithmetic abilities (for ages 11 and 15 only). For premorbid intelligence, the NART was used, measured at age 26 and consisting of the presentation of infrequent words to be read out loud by the participant.

1. Addenbrooke’s Cognitive Examination (ACE-III)

- The ACE-III is a battery of neuropsychological tests^24^ measuring cognitive functions in five domains: (1) attention and orientation, (2) verbal fluency, (3) memory, (4) language and (5) visuospatial function. The total score of each domain was used. ACE-III test scores were assessed at ages 68–70.

1. Socioeconomic status (SES)

- Three variables were used as SES indicators, namely (1) the highest education level reached at 26 years (five categories: no qualification, vocational only, O-level or equivalent, A-level or equivalent, higher education), (2) the overall social class measured by the type of employment level from ages 26, 36 and 43 (six categories: professional, intermediate, skilled (non-manual), skilled (manual), partly unskilled, unskilled) and (3) the child social class measured by the employment level of the main income earner (same categories as social class). For this purpose, the values of age 11 or, if not available, age 15 or 4 were used. All social class measures were coded inversely so that a higher class is represented by a lower number.

1. Health indexes

- We included several self-reported physical health variables, that is, the most important medications (med., ie, antipsychotic med., sedatives, benzodiazepines, antiepileptic med., central nervous system (CNS) med., antidepressants, antiparkinsonian med., neuromuscular relaxants), as well as body mass index (BMI), exercise level (3 categories: none, 1–4 times, 5 or more times a month) and smoking status (3 categories: current smoker, ex-smoker, never smoked), all assessed at ages of 60 and 64.

While psychometric properties for the tasks used in the MRC NSHD data cohort have not been systematically evaluated, it is important to note that many of these tasks have been extensively studied in previous research, which has established their reliability and validity. The LCT exhibits high test–retest reliability (*r*=0.93) and displays strong correlations with other assessments of IPS, such as the Trail Making Test Part A and WAIS Digit Symbol Test, affirming its convergent validity.^25^

The SRT and CRT tasks used in the current study are similar to the computerised Deary-Liewald RTTs.^26^ In a healthy sample aged 18–80, these tasks demonstrated high reliability, with Cronbach’s alpha values of 0.94 for SRT and 0.97 for CRT on correct responses.^26^ Additionally, in healthy older adults, the SRT showed moderate relative variability (interclass coefficient=0.61) while the CRT exhibited good relative variability (interclass coefficient=0.89).^27^

Cognitive assessment batteries used in the MRC NSHD data cohort also show good psychometric properties. The ACE-III has high test–retest reliability for the overall score (*r*=0.90) and individual dimensions (*r*=0.89−0.93),^28^ along with strong internal reliability (Cronbach’s alpha=0.88).^29^ Moreover, the NART demonstrates high construct validity, as evidenced by its loading on general cognitive ability at 0.85.^30^ Regarding reliability, the NART exhibits high internal consistency (Cronbach’s alpha=0.90), as well as excellent test–retest reliability (*r*=0.98)^31^ and inter-rater reliability (*r*=0.88).^32^ Additionally, restandardisation against the WAIS-IV indicates robust correlations between NART scores and premorbid IQ scores.^33^

### Preprocessing

To ensure all variables were weighted equally in the model, we preprocessed numerical variables to keep them in a similar range. For each participant, their scores of verbal memory, intelligence, ACE-III, SES and BMI were divided by 10. LCT scores were divided by 100; SRT and CRT scores were converted from milliseconds to seconds. This approach maintains the interpretability of the path coefficients while reducing model convergence issues due to large differences in variable variances. We then examined the correlations of all variables (see online supplemental table 1) to conceptualise our measurement models and ensure that latent variables were well defined.

Unsurprisingly, SRT and CRT correlated strongly while LCT only correlated moderately with both RTTs, indicating LCT to represent a different type of IPS than SRT and CRT. We, thus, used LCT performance as a measure of cognitive IPS, and the SRT and CRT tasks as measures of motor IPS.

Notably, both RTTs include cognitive components, just as LCT includes motor components. Thus, we cannot claim that LCT exclusively measures cognitive and SRT and CRT exclusively measure motor IPS; new task designs would have to be developed for this. However, using SEM, we were able to form latent constructs based on the shared variance between the measured variables. While cognitive processes are required to different degrees for SRT and CRT, their high correlation should reflect their similarity in motor responses.

Since our latent motor IPS variable is constructed from both RTTs, it should thus reflect motor rather than cognitive processes. Cognitive IPS, solely modelled by LCT, still includes some motor responses, but primarily represents cognitive processes that are not represented by the latent motor IPS variable.

### Statistical analyses

We used SEM in lavaan (R package)^34^ to test how cognitive and demographic measures relate to cognitive and motor IPS, introduced as latent variables. Mardia and Henze-Zirkler tests showed that the data did not form a multivariate normal distribution; thus, we used maximum likelihood with robust SEs to estimate the latent constructs and the path coefficients of our two models.

**Figure 2:**
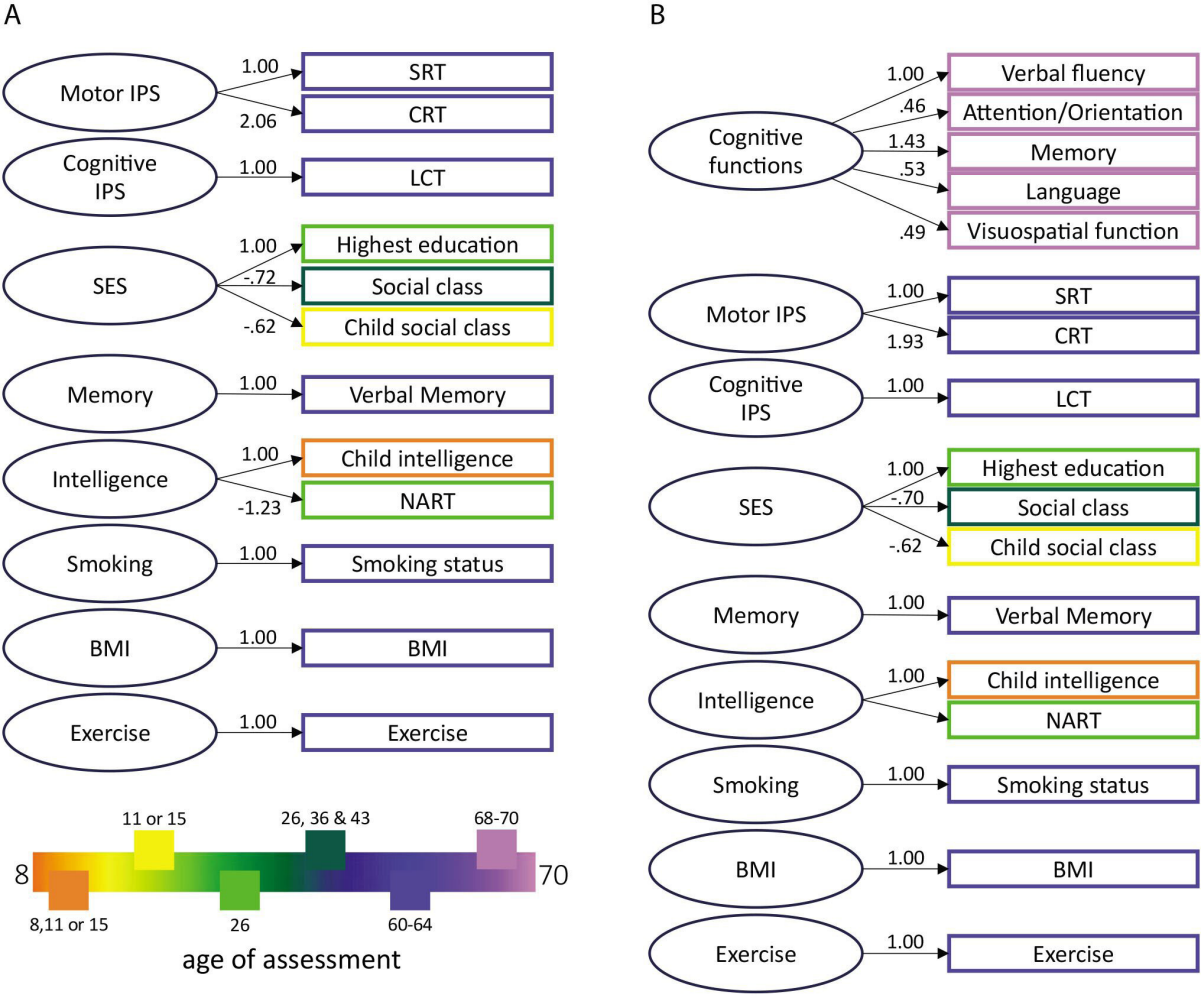
The measurement models for the SEM model 1 (**A**) and model 2 (**B**) with factor loadings. The legend refers to the age of assessment of the respective coloured variables. BMI, body mass index; CRT, choice reaction time; LCT, letter cancellation test; NART, National Adult Reading Test; SEM, structural equation modelling; SES, socioeconomic status; SRT, simple reaction time.

Model 1 aimed to explore the predictors of cognitive and motor IPS measured at ages 60–64. Model 2 explored the indicative value of these two IPS measures on cognitive functions at ages 68–70 (ie, ca. 7 years after the assessment of IPS measures) assessed with the ACE-III battery. For the second model, we only included participants with complete ACE-III measurements, reducing the sample to 1776 cases (932 females). The measurement models for our two models with all latent constructs are shown in figure 2. By constructing latent variables, we ensured that our findings were relevant to the theoretical constructs of interest and not just unique features of certain tests.

Cognitive IPS, memory and confounders (BMI, exercise level and smoking) were included as latent variables with just one predictor as recommended by Hayduk and Littvay.^35^ Their error terms were set to 0 with lavaan’s single predictor option. Binary variables (sex and med. usage) were introduced in the regression of the path analyses (not in the measurement model). Correlations between all latent variables can be found in online supplemental tables 2 and 3.

The two structural models were constructed as follows:

Model 1: cognitive/motor IPS=SES+sex+intelligence+memory+smoking+BMI+exercise+CNS med.+benzodiazepines+antipsychotic med.+antidepressants+antiepileptic med.+antiparkinsonian med.+neuromuscular relaxants+ sedatives.

Model 2: cognitive functions=motor IPS+cognitive IPS+SES+sex+intelligence+memory+smoking+BMI+exercise+CNS med.+benzodiazepines+antipsychotic med.+antidepressants+antiepileptic med.+anti-parkinsonian med.+neuromuscular relaxants+sedatives.

Missing variables were estimated with the maximum likelihood function, as recommended^36 37^ (see online supplemental table 4 for a number of missing variables). Model fits were evaluated using five criteria (1) robust Comparative Fit Index (CFI) and (2) robust Tucker Lewis Index (TLI), both comparing the model to a baseline model (TLI also considering df) with values >0.9 considered acceptable and >0.95 considered good^38^; (3) Root Mean Square Error of Approximation (RMSEA) and (4) Standardised Root Mean Square Residual (SRMR), absolute measures of fit, with values <0.08 considered acceptable and <0.06 considered good.^39^ The *χ*^*2*^ is provided for reference but is not used as a fit measure due to its sensitivity to sample size^40^ and assumption of multivariate normality, which can lead to the rejection of models not meeting this criterion.^41^ Nevertheless, for completeness, we report (5) *χ*^*2*^/df as a normalised measure of relative fit independent of sample size, with smaller values indicating better fit and a cut-off of 5 being a common benchmark.^39^

As commonly used in SEM research, we use the term ‘predict’ in this study to interpret the results. While this denotes that a variable is a strong marker for another, it is important to clarify that it signifies an observed statistical relationship and should not be construed as implying causality.

## Results

When interpreting the results, it is worth noting that SRT and CRT measure reaction times, that is, lower times refer to greater IPS while LCT measures the number of crossed-out words, that is, a greater number means greater IPS. Thus, contrary directions of cognitive and motor IPS in the models and correlations show a relation in the same direction with the variable.

We first constructed the latent variables for our two models within their respective measurement models (the factor loadings of all variables are shown in figure 2). The measurement models for both model 1 and model 2 demonstrated good fit indexes (measurement model for our model 1: *χ*^*2*^ (31)=62.611, *p*<0.001; *χ*^*2*^/df=2.200; RMSEA=0.022; SRMR=0.014; CFI=0.994; TLI=0.987; measurement model for our model 2: *χ*^*2*^ (88)=226.092, *p*<0.001; *χ*^*2*^/df=2.570; RMSEA=0.031; SRMR=0.023; CFI=0.975; TLI=0.961). The factor loadings for our latent variables are presented in figure 2.

**Figure 3:**
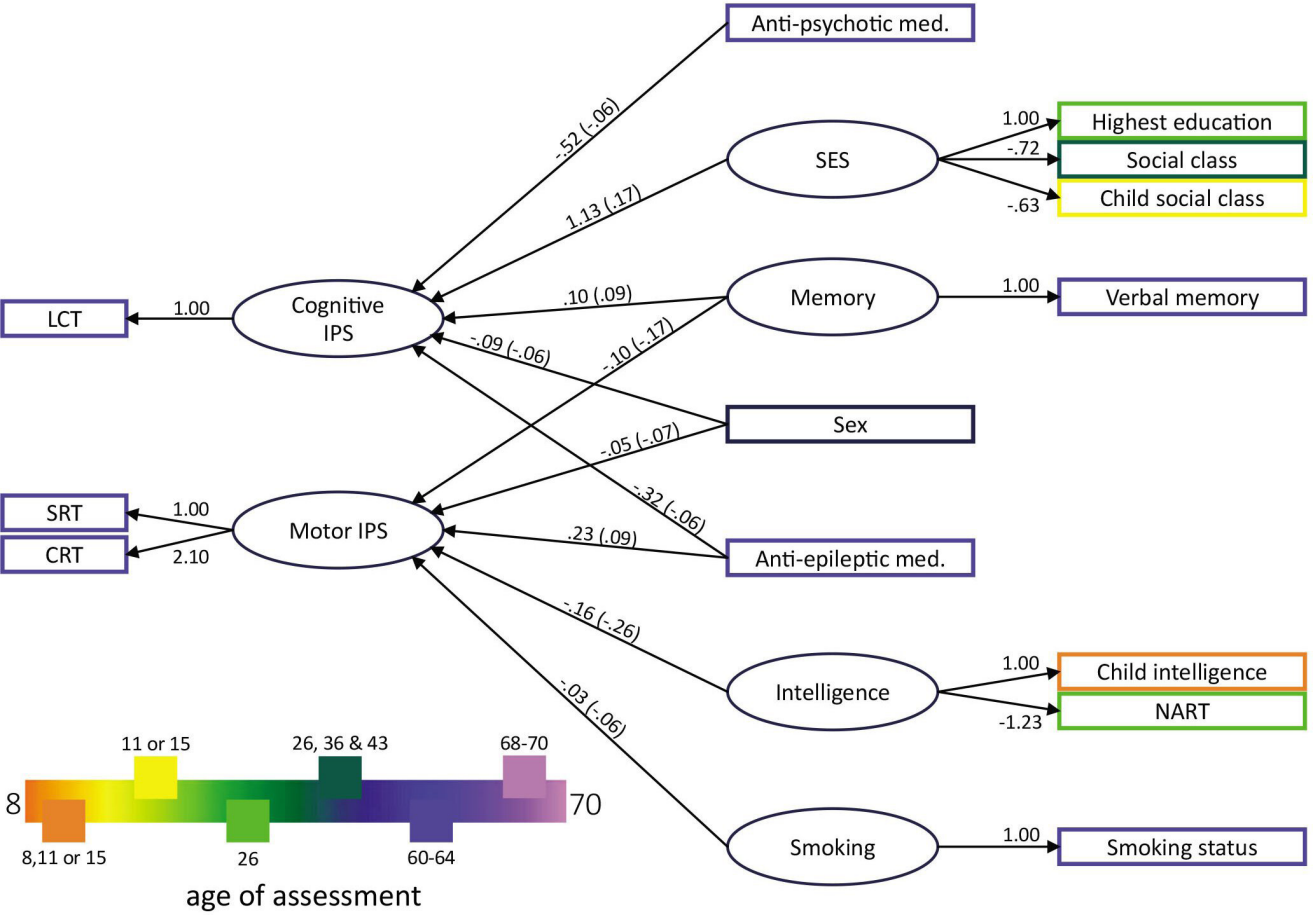
Factor loadings, path coefficients and standardised parameters of the SEM model 1. All latent variables with their loadings and all significant predictors of cognitive and motor IPS from model 1 are shown. The values on the paths indicate unstandardised parameter estimates and standardised parameters (standardised for latent and observed variables) are noted in parenthesis. The legend refers to the age of assessment of the respective coloured variables. CRT, choice reaction time; IPS, information processing speed; LCT, letter cancellation test; Med, medication; NART, National Adult Reading Test; SEM, structural equation modelling; SES, socioeconomic status; SRT, simple reaction time.

Following, we introduced the regression terms within the structural models and estimated the overall SEM models 1 and 2. Model 1 (entailing measurement and structural model) shows good absolute and comparative fit (*χ*^*2*^(131)=521.491, *p*<0.001; *χ*^*2*^/df=3.981; RMSEA=0.038; SRMR=0.031; CFI=0.932; TLI=0.904). Figure 3 displays the factor loadings and path coefficients for all significant predictors. Sex, memory and antiepileptic med. usage emerged as common predictors for both IPS types. Further, motor IPS was predicted by intelligence and smoking while cognitive IPS was predicted by SES and antipsychotic med. usage. Of note, sex was inversely related to both concepts, with women showing greater cognitive and men greater motor IPS. Moreover, these results need to be interpreted while considering the correlation matrix (see online supplemental table 1). For instance, in model 1, SES but not intelligence emerged as a significant predictor for cognitive IPS (as shown in figure 3). However, LCT and both intelligence measures are significantly related with *r*=|0.16|. Due to the nature of SEM, intelligence is not significant in model 1 as it does not explain any further variance of cognitive IPS beyond that what SES already explains.

**Figure 4:**
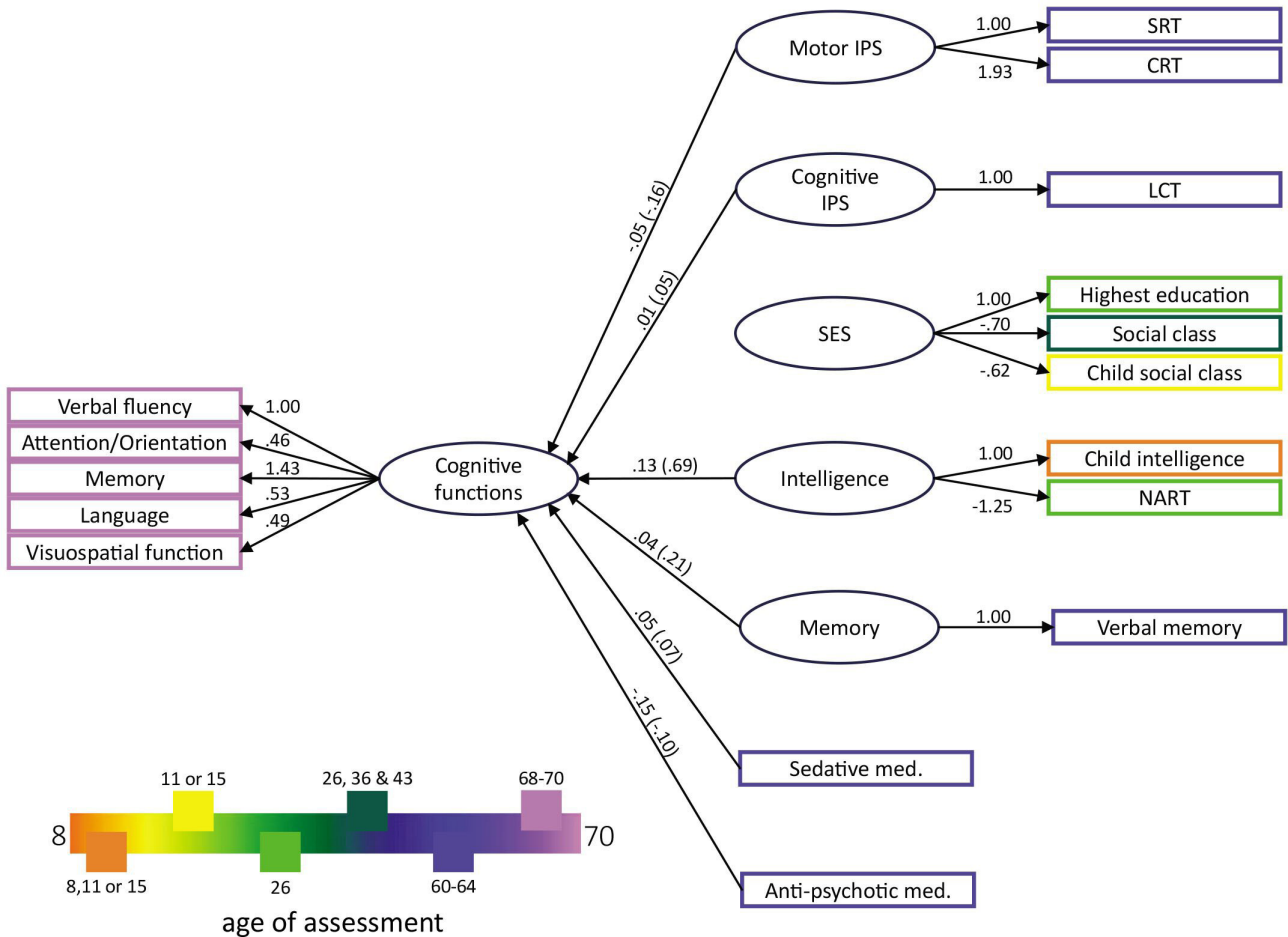
Factor loadings, path coefficients and standardised parameters of the SEM model 2. All latent variables with their loadings and all significant predictors of cognitive functions measured at ages 68–70 from model 2 are shown. The values on the paths indicate unstandardised parameter estimates and standardised parameters (standardised for latent and observed variables) are noted in parenthesis. The legend refers to the age of assessment of the respective coloured variables. CRT, choice reaction time; IPS, information processing speed; LCT, letter cancellation test; Med, medication; NART, National Adult Reading Test; SEM, structural equation modelling; SES, socioeconomic status; SRT, simple reaction time.

Model 2 (entailing measurement and structural model) also shows a good absolute and comparative fit (*χ*^*2*^ (248)=739.414, *p*<0.001; *χ*^*2*^/df=2.982; RMSEA=0.034; SRMR=0.032; CFI=0.918; TLI=0.899). Both IPS measures, as well as intelligence, memory, antipsychotic and sedative med. usage, significantly predicted cognitive functions measured 7 years later (see figure 4 for the factor loadings and path coefficients of all significant predictors). A full overview of the coefficients, including those of non-significant predictors, can be found in online supplemental tables 5 and 6 for models 1 and 2, respectively.

To ensure that the imputation of missing data does not cause biased results, we repeated the analyses of the models only with complete data sets; that is, 1600 cases for model 1 (75% of the participants of the previous model) and 1156 cases for model 2 (65% of the participants of the previous model). Results from model 1 largely remained the same, with two exceptions: (1) SES was no longer significant for cognitive IPS and (2) antiparkinsonian med. was the only significant predictor among all medications. For model 2, cognitive IPS was a weaker and no longer significant predictor. These additional analyses indicate that our main results are robust to the imputation of missing data. Details of these analyses can be found in online supplemental figure 1.

## Discussion

This study reported that, among older adults, cognitive IPS (measured by the LCT) and motor IPS (measured by RTTs) are two constructs that are predicted by different cognitive and demographic variables. Both, cognitive and motor IPS, overlap in having memory, sex and antiepileptic med. as common predictors. Motor IPS is further predicted by intelligence and smoking, and cognitive IPS is further predicted by SES. Conducting a second SEM analysis, we observed that cognitive and motor IPS predict cognitive functions (measured by the ACE-III) 7 years later.

It is important to note that, although some variables have pairwise correlations with cognitive or motor IPS, they are not significant predictors in the SEMs (eg, exercise, BMI, CNS med., benzodiazepines, antidepressants, antiparkinsonian med. and neuromuscular relaxants). This is because such variables do not explain any further variance of the endogenous variables that is not already explained by other predictors or latent variables. Below, we focus our discussions on the significant variables from both SEMs.

### Cognitive and motor IPS measures differ at the theoretical and neural level

At the theoretical level, cognitive IPS can be defined as the rate at which perceptual and automatic cognitive tasks are executed. Cognitive IPS is commonly tested in tasks requiring a sustained level of attention under time constraints.^42 43^ The LCT from the MRC NSHD requires participants to detect and cross out specific letters, entailing the memorisation of target letters and the discrimination of such against distractor letters. This puts a high focus on cognitive abilities, linguistic or visuospatial abilities and lower-order abstraction skills.^44^ Motor IPS as assessed by RTTs, on the other hand, focuses on the efficiency of speeded motor responses.^45^ Contrary to the LCT, the behavioural responses in RTTs primarily involve simple motor processes (ie, pressing specific buttons with specific fingers), from which reaction times statistics can be measured.

There are cognitive components in both SRT and CRT, as well as motor components in LCT. Both RTTs involve visual scanning, discriminating the displayed stimuli and deciding on the appropriate motor response. Moreover, in CRT, participants must decide not only when to make a response but also which response to make (ie, make a choice among response options). Therefore, CRT involves more extensive cognitive processes than SRT, which is reflected in the higher correlation of LCT with CRT than with SRT (see online supplemental table 1). Conversely, LCT captures visuomotor processes as letters must be scanned across the paper (as opposed to constant eye fixation on the screen in computerised RTTs) and hand–eye coordination as letters are crossed.

Nevertheless, it is important to point out that the motor IPS variable in the current study is formed by the common variance of CRT and SRT, which may be primarily related to the motor components of the two tasks, which are different from those in the LCT.

At the neural level, motor and cognitive IPS tasks differ in the extent to which they require secondary association areas (involved in perceptual analysis and synthesis, object recognition, (non-)linguistic analysis and preparation of motor actions).^44^ Cognitive IPS, in particular high LCT performance, is related to increased activity in the middle frontal gyrus, and reduced activity in the cerebellum.^46^ Better performance in motor IPS, as measured by SRT, was previously found to be mostly associated with activation in the occipital lobes, sensorimotor cortices and supplemental motor cortices^47^ along the sensorimotor pathway.^48^

Altogether, our findings confirm the theoretical distinction between cognitive and motor IPS. While common predictors reflect the shared variance of motor and cognitive IPS (such as sensation and perception), distinct predictors underlie the theoretical and neural differences stated above.

### Demographic factors: sex and SES

Model 1 showed women to perform better than men in cognitive IPS and vice versa in motor IPS tasks, which might explain previous heterogeneous research, as stated earlier. Further studies consistently showed that females outperform males in tests involving digits and rapid naming tasks, thus in cognitive IPS tasks. This may be due to sex differences in phonological coding involved in speech-based processes (ie, reading and writing abilities; for a systematic review see^49^). Specifically, sex differences in performance were detected in tasks with letters(-strings),^50 51^ but not in tasks involving geometrical figures.^16^ Consistent with our results, O’Shea *et al* found women to perform higher in the LCT.^52^

Considering motor IPS, research using the FTT^17^ (for a review, see Mitrushina *et al*^53^) and RTTs consistently showed that men are faster than women,^18 19^ consistent with the results in the current study. According to Reimers and Maylor, variability in RTTs possibly reflects sex differences in the trial-to-trial speed-accuracy trade-off, as they found that women were slower and more accurate than men at the beginning but became faster later in test sessions.^54^ However, as the SRT and CRT tasks in the MRC NSHD dataset consisted of a small number of trials with no trial-to-trial data, we were not able to confirm this proposition in the current study.

In a study of the Aberdeen Birth Cohort (ABC) of 1936, SES, composed of parental and own occupational status, predicted late-life IPS.^55^ However, they also found that education and childhood ability were better predictors. Similarly, we found that SES, including parental and own occupational status as well as education level, could predict cognitive IPS and that childhood intelligence is a better predictor for motor IPS. The high covariance between our intelligence and SES latent variables highlights the close relationship and supports the shared variance described by Staff *et al*.^55^

The difference in the relationship between our IPS measures and SES may, again, be a result of the different tasks. The DSST used in the ABC is similar to the LCT but does not involve linguistic processing. As in the ABC study, other research found a correlation between the DSST and SES.^12 56^ Contrastingly, in the English Longitudinal Study of Aging, SES was not correlated with cognitive IPS, measured with the LCT at age 63, but a greater decline in cognitive IPS over time was found to be associated with lower SES.^57^ The differences in how SES was modelled may have led to the distinct results. Nevertheless, we can conclude that this study is in line with our findings, as the relation between SES and decline in cognitive IPS was negative, pointing to a negative impact of lower SES on cognitive IPS.

### Cognitive factors: intelligence and memory

Faster IPS has been related to higher intelligence, especially better g-factor or general intelligence scores.^2 58^ In a previous study, FTT performance was positively correlated to intelligence as measured by the Raven’s Progressive Matrices, WAIS-III verbal IQ and full-scale IQ.^17^ This is in line with our results as we found that motor IPS was predicted by the latent variable intelligence in model 1. Further, studies on the LBC 1936 confirmed that SRT and CRT are moderately related to childhood and premorbid intelligence, measured with the NART,^9 10^ just as in this study. Other research on this cohort also showed, that RTTs were related to general intelligence and that this correlation becomes more pronounced as the number of response options increases.^7 8^ This, again, is in line with our results, as the correlations of child and premorbid intelligence with CRT are greater than with SRT.

In this way, we replicated previous findings of the association between motor IPS and intelligence. However, our cognitive IPS variable measured with the LCT shows a lower correlation with intelligence variables and is not predicted by the intelligence latent variable in our model. Thus, our results point to different characteristics between motor and cognitive IPS. In fact, less work was done on the relationship between cognitive IPS and intelligence and results are heterogeneous with some studies not finding a direct correlation to intelligence at all.

In previous literature, IPS was mostly related to working memory, which is different to short-term memory measured in the MRC NSHD cohort.^59^,^62^ The research measuring short-term memory found moderate correlations between memory tasks and some of the IPS measures.^62^,^64^ For instance, Dang *et al*^62^ and Colom *et al*^64^ found that a number-crossing task, similar to the LCT, had moderate correlations with visuospatial memory and low correlations with verbal memory. This may be due to the domain of the tasks, with a greater relation of visuospatial to numeric than to verbal abilities. Conway *et al* found that IPS, measured with a simple pattern and letter comparison task, was moderately correlated to verbal memory, but only in an articulatory suppression condition.^63^ These two studies used several IPS, short-term memory and working memory tasks confirming that working memory shows a closer relation to IPS than short-term memory. However, with great differences in the instruments used and heterogeneity in the results of each test, it is difficult to draw conclusions on how strong the relation between the different types of IPS and memory is.

### Health factors: smoking and medication

Although the immediate effect of smoking, that is, high levels of nicotine, has been related to better cognitive functions,^65^ long-term smokers have shown worse performance in cognitive tasks in later life.^66^,^68^ For instance, a faster decline in memory in later life was found in both the MRC NSHD and ABC cohorts.^67 68^ In terms of IPS measures, visual search speed at age 43 was reported to be decreased in smokers^67^ and the DSST also showed lower performance in smokers.^68^ However, in general, effect sizes have been low, just as in our study. We found smoking as a predictor for motor IPS but not cognitive IPS. Heterogeneous findings may be due to age differences at the time of data collection and in the design of the statistical models, as the raw correlations of all IPS measures with smoking status are low.

We found that antiepileptic, antipsychotic and sedative med. were significant predictors of IPS. Previous research found that epilepsy is linked to white matter loss and slower reaction times,^69 70^ and some antiepileptic drugs have been reported to affect cognition and IPS.^71^ In psychotic disorders, it is known that most patients already have cognitive deficits before the first psychotic episode.^72^ More specifically, schizophrenia is associated with IPS impairments using the DSST, as shown by a meta-analysis.^73^

Sedatives have also been associated with cognitive impairment. Depending on the drug, different cognitive processes are affected, for example, memory,^74^ as well as attention and psychomotor functions.^75^ In our study, however, we found a positive effect of sedatives on cognitive functions measured in later life. This may be attributed to the diverse reasons for using these drugs, as they are often prescribed for sleep disorders and may not be directly linked to diseases involving cognitive impairment.

### IPS as a predictor of cognitive functions in later life

Model 2 explored understanding the temporal dynamics of motor and cognitive IPS on cognitive functions 7 years later. Our results showed that later-life cognitive functions, measured by the ACE-III at ages 68–70, are predicted by both motor and cognitive IPS. There is a large body of research on the cross-sectional association between IPS and cognitive functions,^76^,^78^ but only a few investigated longitudinal effects. Finkel *et al* applied growth curve modelling of IPS and cognitive functions, showing that faster IPS relates to slower deterioration in spatial and memory performance.^79^ Taken together, our results are in line with the general notion that IPS is strongly associated with cognitive functioning.

In the current study, LCT was referred to as a cognitive IPS measure, and response times from the SRT and CRT tasks were referred to as motor IPS measures. Interestingly, motor IPS has a higher path coefficient with cognitive functions compared with cognitive IPS (see figure 4). This result appears to be counterintuitive, and it may stem from the specificity of the LCT. While the LCT involves executive functions, the execution of the LCT primarily involves visual processing and selective attention.^80 81^ Hence, the specific cognitive demand required by the LCT may constrain the task’s associations with a broader range of cognitive functions. For example, no correlation was found between the LCT performance and verbal IQ.^25^ Furthermore, LCT performance depends on visual working memory,^82^ which could account for the weak correlation with memory measures observed in our study (online supplemental tables 1–3).

On the other hand, reaction times from the SRT and CRT tasks, which quantify motor IPS, are also strongly associated with cognitive functioning and intelligence. Faster motor responses are linked to higher intelligence scores. Sheppard and Vernon^8^ further showed a stronger correlation between RTT performance with fluid and crystallised intelligence than memory processing tasks with intelligence. Hence, our results and previous findings highlight the close relationship between simple motor IPS measures and cognitive ability.

Due to the MRC NSHD data set’s limitations, we were unable to construct our cognitive IPS variable with more tests other than the LCT. Future research incorporating a wider range of cognitive IPS measures could provide deeper insights into these relationships, potentially reducing the observed differences between the path coefficient of motor IPS with cognitive functioning and that of cognitive IPS.

Nevertheless, these findings have implications for understanding the stability of cognitive abilities over time. The factors captured by motor and cognitive IPS seem to be important contributors to cognitive abilities that remain consistent over a 7-year period. This could suggest that interventions or strategies targeting these factors (eg, in the context of cognitive training) might have the potential to lead to long-term improvements in cognitive abilities.

### Implications and future directions

IPS measures, such as the LCT and RTTs, as well as the Colour Trails Test and DSST, are frequently used in clinical settings for the diagnosis of patients. Our results show that the performance in such tests cannot be interpreted as a single IPS construct because cognitive and motor IPS relate to different cognitive and demographic factors. We used data from the MRC NSHD cohort, allowing us to base our findings on a large longitudinal cohort. As all waves include a medical and cognitive assessment, we were able to consider confounder variables, such as med. usage and smoking status. This allowed us to design SEM analyses with important predictors of IPS, as well as other variables that could have influenced the measurements. However, two issues require further consideration. First, we were constrained by the available variables in the MRC NSHD dataset, in which other forms of IPS measures (eg, DSST) were not assessed. Second, motor IPS was estimated from a limited number of trials as part of a complicated protocol. As a result, we could not apply cognitive modelling for motor IPS^83^ beyond simple RT statistics. Future studies should consolidate our findings in other cohort datasets and IPS tasks.

### Conclusion

In conclusion, our results support a multifaceted understanding of IPS, revealing that motor and cognitive IPS are influenced by distinct variables in older adults. Specifically, motor IPS serves as a robust predictor of cognitive functions in later life, whereas cognitive IPS is a comparatively weaker predictor. These results validate the use of IPS as a fundamental marker of intelligence and cognition, though it is crucial to consider differences in task design.

## supplementary material

10.1136/bmjopen-2024-083968 online supplemental file 1

## Data Availability

Data may be obtained from a third party and are not publicly available.
