# Supplementary material for: Characterising and differentiating cognitive and motor speed in older adults: structural equation modelling on a UK longitudinal birth cohort
Source: BMJ Open. 2024 Aug 19;14(8):e083968. doi: 10.1136/bmjopen-2024-083968 (PMC11337668; doi:10.1136/bmjopen-2024-083968)
Supplement: online supplemental file 1 [file bmjopen-14-8-s001.pdf]

Characterising and differentiating cognitive and motor speed in older adults: Structural Equation Modelling on a UK longitudinal birth cohort

Indra Bundil[a,*], Sabina Baltruschat[a,*], and Jiaxiang Zhang[a,b]

a. School of Psychology, Cardiff University, Cardiff, UK

b. Department of Computer Science, Swansea University, Swansea, UK

* These two authors contributed equally to this work.

Corresponding Author:

Indra Bundil (bundilIM@cardiff.ac.uk)

Supplementary Table 1. Correlations between all variables used in SEMs

	1 SRT	2	3	4	5	6	7	8	9	10	11	12	13	14	15	16
**2 CRT**	.50**															
**3 LCT**	-.14**	-.25**														
**4 Education**	-.17**	-.26**	.16**													
**5 SES**	.16**	.24**	-.14**	-.50**												
**6 Child SES**	.15**	.18**	-.12**	-.38**	.29**											
**7 NART**	.21**	.31**	-.16**	-.54**	.42**	.35**										
**8 Child intelligence**	-.18**	-.29**	.16**	.52**	-.41**	-.36**	-.66**									
**9 Memory**	-.20**	-.31**	.19**	.39**	-.33**	-.27**	-.48**	.46**								
**10 ACE fluency**	-.25**	-.29**	.18**	.27**	-.21**	-.21**	-.38**	.37**	.37**							
**11 ACE language**	-.18**	-.19**	.10*	.28**	-.25**	-.23**	-.44**	.38**	.29**	.30**						
**12 ACE attention**	-.08*	-.09**	.06*	.17**	-.15**	-.08*	-.17**	.19**	.18**	.20**	.13**					
**13 ACE memory**	-.14**	-.22**	.16**	.30**	-.26**	-.23**	-.41**	.36**	.47**	.30**	.35**	.16**				
**14 ACE visuospatial**	-.17**	-.24**	.11**	.30**	-.23**	-.21**	-.30**	.30**	.27**	.26**	.25**	.20**	.30**			
**15 Exercise**	-.08**	-.11**	.08**	.18**	-.18**	-.14**	-.17**	.17**	.19**	.11**	.09**	.06*	.12**	.09**		
**16 Smoking**	-.02	-.12**	.09**	.15**	-.13**	-.08*	-.11**	.07*	.14**	.05	.06*	-.02	.13**	.04	.12**	
**17 BMI**	.06*	.07*	-.07*	-.12	.10	.16	.11	-.08	-.12	-.07	-.05	-.02	-.07	-.09	-.11	<.01

Note. *P*-values are highlighted as * < .05; ** < .001. ACE: Addenbrooke's Cognitive Examination; BMI: Body Mass Index; CRT: Choice Reaction Time; LCT: Letter Cancellation Test; NART: National Adult Reading Test; SES: Socio-economic status; SRT: Simple Reaction Time.

Supplementary Table 2. Correlations of latent variables for Model 1

	Motor IPS	Cognitive IPS	SES	Intelligence	Memory	Smoking	BMI
**Cognitive IPS**	-.3**						
**SES**	-.476**	.249**					
**Intelligence**	-.502**	.218**	.932**				
**Memory**	-.402**	.199**	.582**	.646**			
**Smoking**	-.142**	.093**	.223**	.129**	.148**		
**BMI**	.084**	-.067*	-.199**	-.132**	-.122**	.003	
**Exercise**	-.142**	.081*	.289**	.235**	.191**	.132**	-.109**

Note. * < .005; ** < .001

Supplementary Table 3. Correlations of latent variables for Model 2

	Motor IPS	Cognitive IPS	SES	Intelligence	Memory	Smoking	BMI	Exercise
**Cognitive IPS**	-.324***							
**SES**	-.528***	.228***						
**Intelligence**	-.586***	.212***	.933***					
**Memory**	-.441***	.177***	.574***	.632***				
**Smoking**	-.166***	.082***	.22***	.124***	.133***			
**BMI**	.101***	-.056*	-.196***	-.116***	-.114***	-.026		
**Exercise**	-.121***	.061*	.267***	.209***	.168***	.129***	-.095***	
**Cognitive functions**	-.674***	.292***	.834***	.924***	.734***	.146***	-.129***	.215***

Note. * < .05; ** < .005; *** < .001

Supplementary Table 4. Number of missing data for individual variables, separately for Model 1 and Model 2

	**Model 1** (*N*=2124)	**Model 2** (*N*=1776)
**CNS med.**	1	-
**Anti-psychotic med.**	1	-
**Anti-depressants**	1	-
**Anti-epileptic med.**	1	-
**Anti-parkinsonian med.**	1	-
**Sedatives**	1	-
**Highest education level**	18	12
**Overall social class**	11	6
**Child social class**	109	93
**Exercise**	63	45
**Smoking**	191	127
**BMI**	3	2
**Child IQ**	147	123
**NART**	158	108
**Memory**	38	19

Note. BMI: Body Mass Index; Med: Medication; NART: National Adult Reading Test.

Supplementary Table 5. Regressions for Model 1

	Estimate	*SE*	*z*	*p*	Std.lv	Std.all
**Motor IPS ~**						
SES	-.205	.281	-.731	.465	-.059	-.059
Sex	-.053	.016	-3.222	.001	-.141	-.070
Intelligence	-.155	.052	-2.965	.003	-.263	-.263
Memory	-.104	.023	-4.576	<.001	-.171	-.171
Smoking	-.032	.015	-2.073	.038	-.058	-.058
BMI	.001	.018	.045	.964	.001	.001
Exercise	-.006	.010	-.629	.530	-.014	-.014
CNS med.	.025	.032	.794	.427	.067	.027
Benzodiazepines	-.018	.120	-.146	.884	-.047	-.006
Anti-psychotic med.	-.091	.172	-.530	.596	-.244	-.021
Anti-depressants	.052	.050	1.038	.299	.139	.038
Anti-epileptic med.	.234	.083	2.812	.005	.624	.086
Anti-parkinsonian med.	.019	.201	.095	.924	.051	.003
Neuromuscular Relaxants	-.036	.056	-.636	.525	-.096	-.011
Sedatives	.131	.088	1.493	.135	.350	.051

Supplementary Table 5 (continued). Regressions for Model 1

	Estimate	*SE*	*z*	*p*	Std.lv	Std.all
**Cognitive IPS ~**						
SES	1.127	.458	2.462	.014	.172	.172
Sex	-.088	.031	-2.820	.005	-.124	-.062
Intelligence	-.004	.078	-.057	.955	-.004	-.004
Memory	.101	.033	3.070	.002	.087	.087
Smoking	.036	.025	1.439	.150	.034	.034
BMI	-.037	.031	-1.186	.236	-.025	-.025
Exercise	.010	.019	.554	.580	.012	.012
CNS med	.028	.053	.541	.588	.040	.016
Benzodiazepines	-.210	.198	-1.057	.290	-.295	-.035
Anti-psychotic med.	-.520	.263	-1.974	.048	-.731	-.063
Anti-depressants	.038	.068	.560	.576	.053	.015
Anti-epileptic med.	-.315	.110	-2.858	.004	-.443	-.061
Anti-parkinsonian med.	-.430	.290	-1.480	.139	-.604	-.035
Neuromuscular Relaxants	-.063	.105	-.594	.553	-.088	-.010
Sedatives	.072	.167	.428	.669	.101	.015

Note. Columns illustrate path coefficient estimates, standard errors, *z*-values, significance (*p*-) values, coefficient estimates after standardising the latent variable only, and coefficient estimates after standardising all variables. BMI: Body Mass Index; Med: Medication; *SE*: Standard Error; Std.all: coefficients standardised for all variables (observed and latent); Std.lv: coefficients standardised for only latent variables.

Supplementary Table 6. Regressions for Model 2

	Estimate	*SE*	*z*	*p*	Std.lv	Std.all
**Motor IPS**	-.054	.017	-3.188	.001	-.155	-.155
**Cognitive IPS**	.009	.004	2.086	.037	.053	.053
**SES**	-.085	.108	-.784	.433	-.079	-.079
**Sex**	-.005	.006	-.776	.438	-.040	-.020
**Intelligence**	.130	.020	6.432	<.001	.694	.694
**Memory**	.040	.007	6.039	<.001	.210	.210
**Smoking**	.001	.005	.109	.913	.003	.003
**BMI**	.001	.007	.091	.927	.002	.002
**Exercise**	.002	.003	.649	.516	.015	.015
**CNS med**	-.019	.010	-1.843	.065	-.167	-.066
**Benzodiazepines**	-.055	.035	-1.537	.124	-.472	-.056
**Anti-psychotic med**	-.147	.042	-3.465	.001	-1.271	-.104
**Anti-depressants**	.010	.015	.706	.480	.090	.023
**Anti-epileptic med**	-.007	.026	-.286	.775	-.064	-.009
**Anti-parkinsonian med**	-.061	.067	-.905	.366	-.524	-.033
**Neuromuscular Relaxants**	-.042	.030	-1.395	.163	-.363	-.044
**Sedatives**	.054	.023	2.401	.016	.469	.067

Note. Columns illustrate path coefficient estimates, standard errors, *z*-values, significance (*p*-) values, coefficient estimates after standardising the latent variable only, and coefficient estimates after standardising all variables. BMI: Body Mass Index; Med: Medication; *SE*: Standard Error; Std.all: coefficients standardised for all variables (observed and latent); Std.lv: coefficients standardised for only latent variables.

Supplementary Results: SEM analyses without imputing missing data

The analyses of the models only with complete data cases show very similar results with acceptable comparative and good absolute fit measure; Model 1: $\chi^2(131) = 442.473$, $p < .001$; $\chi^2/df = 3.378$; RMSEA = .037; SRMR = .033; CFI = .927; TLI = .896, Model 2: ($\chi^2(248) = 645.878$, $p < .001$; $\chi^2/df = 2.604$; RMSEA = .038; SRMR = .036; CFI = .900; TLI = .876). Supplementary Figure 1 shows the models and the factor loadings and path coefficient.

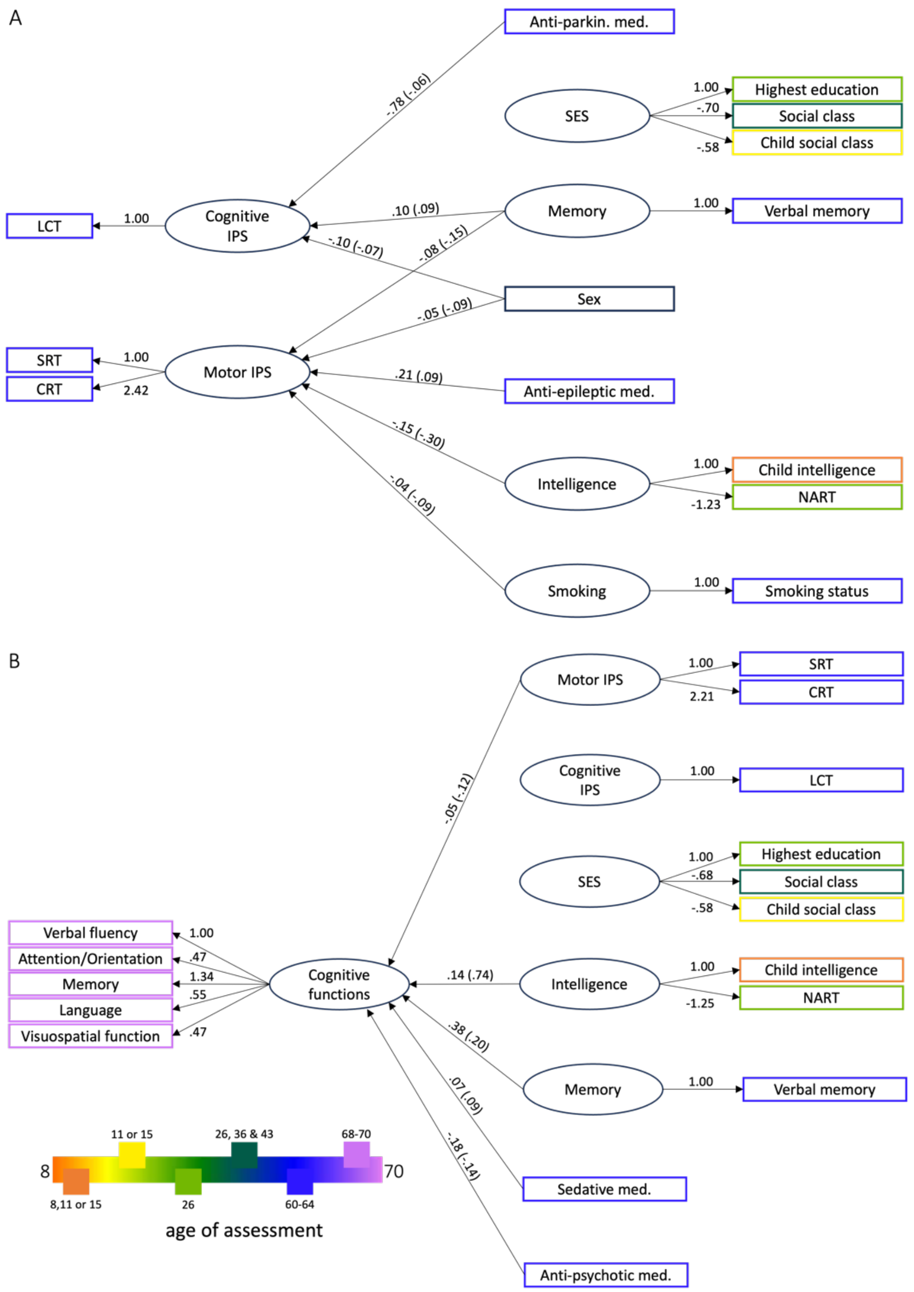

Supplementary Figure 1. Factor loadings, path coefficients and standardised parameters of SEM Models 1 and 2 without imputing missing data. A shows all latent variables with their loadings and all significant predictors of cognitive and motor IPS (Model 1); B shows all latent variables with their loadings and all significant predictors of cognitive functions measures at ages 68-70 (Model 2). Standardised parameters are noted in parenthesis. The legend refers to the age of assessment of the respective coloured variables. CRT: Choice Reaction Time; LCT: Letter Cancellation Test; Med: Medication; NART: National Adult Reading Test; Anti-parkin: Anti-parkinsonian; SES: Socio-economic status; SRT: Simple Reaction Time.
