# Supplementary material for: Characterising and differentiating cognitive and motor speed in older adults: structural equation modelling on a UK longitudinal birth cohort
Source: BMJ Open. 2024 Aug 19;14(8):e083968. doi: 10.1136/bmjopen-2024-083968 (PMC11337668; doi:10.1136/bmjopen-2024-083968)
Supplement: Supplementary file 2 [file bmjopen-2024-083968.reviewer_comments.pdf]

PEER REVIEW HISTORY

BMJ Open publishes all reviews undertaken for accepted manuscripts. Reviewers are asked to complete a checklist review form and are provided with free text boxes to elaborate on their assessment. These free text comments are reproduced below.

ARTICLE DETAILS

**TITLE (PROVISIONAL)**	Characterising and differentiating cognitive and motor speed in older adults: Structural Equation Modelling on a UK longitudinal birth cohort
**AUTHORS**	Bundil, Indra; Baltruschat, Sabina; Zhang, Jiaxiang

VERSION 1 - REVIEW

**REVIEWER NAME**	Roivainen, Eka
**REVIEWER AFFILIATION**	Oulu University Hospital
**REVIEWER CONFLICT OF INTEREST**	None.
**DATE REVIEW RETURNED**	14-Feb-2024

**GENERAL COMMENTS**	This study addresses an important topic: cognitive speed in older adults. The data come from a large sample and the analyses performed by the authors are relevant. I recommend publication of this study with some revisions.<br>1. Introduction, Page 5, line 58. According to the authors Ritchie et al. reported dissociation between IPS and education. I think this is a misunderstanding. I think Ritchie et al. meant the rate of decline or slope:<br>Contrary to some conceptions of 'cognitive reserve' (Stern, 2002), we found no evidence for a relation between education (or social class) and the slope of any of the cognitive factors…. our results were a further test of the previously-asked question regarding whether 'age is kinder to the initially more able' (Gow et al., 2012). The answer, from our fully-adjusted model, is 'no'.(Ritchie et al., 2013)<br>I believe there is a consensus on positive correlation between education and any cognitive skill, including Processing speed. For example, in the benchmark WAIS IV test the PSI index mean was 89 for persons with 8 years or less of education and 106 for persons with >17 years of education in the US standardization sample of n=2250 (Holdnack et al., 2014)<br>The authors could expand their literature search and include more studies reporting the association between the variables studied (speed, IQ, SES, education…).<br>2. Method. Page 6, Variables. The authors could discuss the validity and reliability of the test methods used.<br>3. Results. Table 1. There are some results that seem counterintuitive. For example, correlation between SRT/CRT and education are slightly stronger than that of LCT and education despite LCT being a more complex test involving f ex. executive functions. Likewise, SRT and CRT have a stronger correlation with all ACE tests as compared to LCT/ACE correlations. I suppose that the problem lies with the LCT test.<br>The authors could compare their results with those from other studies using popular cognitive test methods that have analyzed the

	associations between IQ, education, academic skills, Processing speed, reaction time, etc.

**REVIEWER NAME**	Ricciardi, Elisabetta
**REVIEWER AFFILIATION**	University of Bari, Educational Sciences, Psychology and Communication
**REVIEWER CONFLICT OF INTEREST**	None.
**DATE REVIEW RETURNED**	14-Mar-2024

**GENERAL COMMENTS**	The manuscript titled “Characterizing and differentiating cognitive and motor speed in older adults: a longitudinal birth cohort study” attempts to explore Information Processing Speed (IPS) as a multifaceted concept. Specifically, this study aims to explore individual differences in IPS measures, and to investigate the association between IPS and cognitive decline. To this end, two structural equation models were performed using data from the MRC NSHD, a large cohort of UK adults. The present study, with its results addresses new knowledge to issues related to IPS in older age.<br>I have carefully read the manuscript, and I think that it may be of interest for the readers of BMJ Open. However, I also think that several major points need to be addressed to the manuscript. Below there are my comments and suggestions.<br>Introduction<br>The rationale for this study needs improvement. It would be relevant to better explain why this study is useful, identify the research gap it aims to address in the scientific literature, and emphasize why it is important to address this gap. To better highlight the significance of the study, I suggest extending this section.<br><br>Method<br>Cohort data<br>I suggest adding some information (e.g., gender, age means, sd) about the participants resulting available for the present study. Information about ethical statement and informed consent needs to be clarify.<br><br>Variables<br>Even if details on each measure can be found in Moulton et al. I think that may be useful add in this section more information, such as references, validation, and psychometric characteristics, about the assessment measures.<br>Lines 142-146, page 8: Please, specify if health indexes were self-reported or not.<br><br>Pre-processing<br>Data preprocessing was conducted; however, why was standardization not performed?<br><br>Statistical Analysis<br>Lines 181-188, page 10: the model 1 and the model 2 are not clear. For example, I suppose that the model 1 “Cognitive/Motor IPS = Socio-economic status + sex + intelligence + memory + smoking + BMI + exercise + CNS med. + benzodiazepines + anti-psychotic med. + anti-depressants + anti-epileptic med. + anti- parkinsonian med. + neuromuscular relaxants + sedatives” should correspond

	with the figure at page 30. Please, could the Author(s) clarify this point? Also, for the model 2.<br>Lines 189-196, page 10: “Model fits were determined by two measures” but three indexes were reported (i.e., CFI; RMSEA, SRMR). Furthermore, why did the author(s) choose not to report the TLI as a fit measure?<br>Lines 194-196, page 10: As the Author(s) state in the manuscript, due the highly sensitivity to the sample size $\chi2$ is reported only for information. I suggest using the $\chi2/df$ as a fit measure.<br><br>Results<br>Lines 206-207, page 10: "(Model 1: $\chi2(31) = 62.611$, $p < .001$; RMSEA = .022; SRMR = .014; CFI = .994; Model 2: $\chi2$ (88) = 226.092, $p < .001$; RMSEA = .031; SRMR = .023; CFI = .975)"<br>These results do not correspond to the results from Model 1 at page 11, lines 208-219, and Model 2 at page 11, lines 218-219. I suppose that are four different models (two for the measurements models and the other two as main analysis). It seems to be a labeling problem. Could the author(s) fix it?<br>I can’t see tables for main analysis, as well as caption for figure at page 28, 29, and 30. Could the author(s) add it?<br><br>Discussion<br>As far as I am concerned, results were extensively discussed in light of previous studies.

VERSION 1 – AUTHOR RESPONSE

Response to Reviewer 1’s comments

1. Introduction, Page 5, line 58. According to the authors Ritchie et al. reported dissociation between IPS and education. I think this is a misunderstanding. I think Ritchie et al. meant the rate of decline or slope: Contrary to some conceptions of ‘cognitive reserve’ (Stern, 2002), we found no evidence for a relation between education (or social class) and the slope of any of the cognitive factors…. our results were a further test of the previously-asked question regarding whether ‘age is kinder to the initially more able’ (Gow et al., 2012). The answer, from our fully-adjusted model, is ‘no’. (Ritchie et al., 2013)

I believe there is a consensus on positive correlation between education and any cognitive skill, including Processing speed. For example, in the benchmark WAIS IV test the PSI index mean was 89 for persons with 8 years or less of education and 106 for persons with >17 years of education in the US standardization sample of n=2250 (Holdnack et al., 2014) The authors could expand their literature search and include more studies reporting the association between the variables studied (speed, IQ, SES, education…).

We thank the Reviewer for suggesting the two additional references, which we have included in the revised Introduction (lines 68-70 & lines 72-73). Additionally, we have expanded the Introduction, referencing studies that show associations of IPS with Logical Memory, Verbal Fluency, National Adult Reading Test, the Wechsler Test of Adult Reading and Letter-Number Sequencing (WAIS-III), emphasizing the complex relationships between IPS and other cognitive domains (lines 50-55).

First, we would like to clarify the confusion of the two studies: (1) Ritchie et al. (2013) cited in our original manuscript titled “Education is associated with higher later life IQ scores, but not with faster cognitive processing speed”, and (2) Ritchie et al. (2016) suggested by the Reviewer titled “Predictors of ageing-related decline across multiple cognitive functions”.

Ritchie et al. (2013) utilised data from the Lothian Birth Cohorts (LBC) 1921 and 1936, with cognitive abilities assessed at ages 11 (prior to differential education), 70 (LBC 1936), 79 (LBC 1921), and 83 (LBC 1921). IPS was assessed using Simple Reaction Time (SRT), 4-Choice Reaction Time (CRT), and Inspection Time (IT). While some IPS measures showed significant associations with education, these effects vanished when accounting for age 11 IQ prior to divergent educational paths. Furthermore, their results showed a relationship between childhood IQ and education. We have summarised these findings in the Revision (lines 78-84 and see below in this response).

Second, as highlighted by the Reviewer, there is abundant evidence suggesting associations between cognitive skills (including IPS) and education. In the revised Introduction, we start out by referencing studies that examined such associations, including the two papers mentioned by the Reviewer (i.e., Holdnack et al., 2013; Ritchie et al., 2016). “On one hand, substantial evidence links cognitive functions, including IPS, to educational attainment. For instance, the WAIS-IV standardisation study reported a mean processing speed index of 86 for individuals with less than 8 years of education, compared to 106 for those with more than 18 years (Holdnack et al., 2013). Zhang et al. (2015) also found significant associations between IPS (measured by the Digit Symbol Substitution Test) and education. On the other hand, a growth curve modelling study showed that the rate of cognitive decline, including IPS, is not associated with educational level (Ritchie et al., 2016)” (lines 67-73).

We build on this by suggesting that these discrepancies “may be due to the focus on a few selected variables for investigation, without accounting for their covariance with additional variables. For instance, Richtie et al. (2013) found that the correlation between IPS (measured via SRT, CRT and IT) and education became non-significant after controlling for childhood IQ, measured prior to differential education at age 11. These findings suggest that while later life IPS is linked to education, childhood IQ (likely indicative of subsequent educational attainment) emerges as the primary determinant. Additionally, this raises the question of whether IPS is independently influenced by education or if observed associations are due to shared variance with intelligence” (lines 77-84).

“Together, the inconsistencies in findings underscore the necessity of not only examining pairwise correlations between variables but also considering their covariances within a statistical model. Consequently, the primary aim of this study is to employ Structural Equation Modelling to delineate the relationships between specific variables, accounting for their covariances with correlated cognitive and demographic factors. To achieve this, we set out with two main objectives: (1) To model individual differences in IPS measures at ages 60-64 (LCT, SRT/CRT) with variables known to be associated with IPS (Model 1) and (2) to investigate the longitudinal association of IPS with cognitive decline at ages 68-70, while controlling for health-related, demographic, and cognitive variables (Model 2).

We utilise data from the Medical Research Council National Survey of Health and Development cohort, which includes measurements similar to those of the LBC 1936. By modelling separate variables to capture cognitive and motor IPS, we aim to better understand their distinct and overlapping components, addressing a gap in the literature where IPS is often treated as a single construct” (lines 106-117).

2. Method. Page 6, Variables. The authors could discuss the validity and reliability of the test methods used.

In the Revision, we have discussed the psychometric properties (i.e., validity and reliability) of RT tasks, the LCT, as well as ACE and NART (lines 202-219):

“While psychometric properties for the tasks used in the MRC NSHD data cohort have not been systematically evaluated, it is important to note that many of these tasks have been extensively studied in previous research, which have established their reliability and validity. The LCT exhibits high test-retest reliability (r = .93) and displays strong correlations with other assessments of IPS, such as the Trail Making test Part A and WAIS Digit Symbol test, affirming its convergent validity (Uttl & Pilkenton-Taylor, 2001).

The SRT and CRT tasks used in the current study are similar to the computerised Deary-Liewald RTTs (Deary et al., 2011). In a healthy sample aged 18-80, these tasks demonstrated high reliability, with Cronbach's alpha values of .94 for SRT and .97 for CRT on correct responses (Deary et al., 2011). Additionally, in healthy older adults, the SRT showed moderate relative variability (Inter-Class Coefficient = 0.61), while the CRT exhibited good relative variability (Inter-Class Coefficient = 0.89) (Ferreira et al., 2021).

Cognitive assessment batteries used in the MRC NSHD data cohort also show good psychometric properties. The ACE-III has high test-retest reliability for the overall score (r = .90) and individual dimensions (r = .89-.93) (Alilou et al., 2017), along with strong internal reliability (Cronbach's alpha = .88) (Noone, 2015). Moreover, the NART demonstrates high construct validity, as evidenced by its loading on general cognitive ability at 0.85 (Crawford, Stewart, et al., 1989). Regarding reliability, the NART exhibits high internal consistency (Cronbach's alpha = 0.90), as well as excellent test-retest reliability (r = 0.98) (Crawford, Parker, et al., 1989) and interrater reliability (r = 0.88) (O’Carroll, 1987). Additionally, re-standardisation against the WAIS-IV indicates robust correlations between NART scores and premorbid IQ scores (Bright et al., 2018).”

3. Results. Table 1. There are some results that seem counterintuitive. For example, correlation between SRT/CRT and education are slightly stronger than that of LCT and education despite LCT being a more complex test involving f ex. executive functions. Likewise, SRT and CRT have a stronger correlation with all ACE tests as compared to LCT/ACE correlations. I suppose that the problem lies with the LCT test.

The authors could compare their results with those from other studies using popular cognitive test methods that have analyzed the associations between IQ, education, academic skills, Processing speed, reaction time, etc.

We appreciate the Reviewer's comments regarding the strength of correlations observed in our results, particularly on the relationships between different IPS measures and ACE dimensions.

In this context, we wish to clarify our approach to data analysis and interpretation. In our study, the primary objective is not to assess pairwise correlations between variables because, as demonstrated in Supplementary Table 1 (also our response to Point 1), many variables correlate with each other. Instead, our focus lies in utilising Structural Equation Modelling to examine the complex contributions to cognitive and motor IPS measures from multiple variables. We acknowledge that this counterintuitive finding also becomes apparent when looking at the standardised path coefficients where motor IPS seems more strongly related with cognitive function than cognitive IPS within our SEM Model 2.

Therefore, to further address the Reviewer's comment, we have extended the Discussion in the Revision (lines 466-485). "In the current study, LCT was referred to as a cognitive IPS measure, and response times from the SRT and CRT tasks were referred to as motor IPS measures. Interestingly, motor IPS has a higher path coefficient with cognitive functions compared to cognitive IPS (see Figure 4). This result appears to be counterintuitive, and it may stem from the specificity of the LCT. While the LCT involves executive functions, the execution of the LCT primarily involves visual processing and selective attention (Hatta et al., 2012; Saito et al., 2015). Hence, the specific cognitive demand required by the LCT may constrain the task's associations with a broader range of cognitive functions. For example, no correlation was found between the LCT performance and verbal IQ (Uttl & Pilkenton-Taylor, 2001). Furthermore, LCT performance depends on visual working memory (Treviño et al., 2021), which could account for the weak correlation with memory measures observed in our study (Supplementary Tables 1, 2, and 3).

On the other hand, reaction times from the SRT and CRT tasks, which quantify motor IPS, are also strongly associated with cognitive functioning and intelligence. Faster motor responses are linked to higher intelligence scores (Deary et al., 2001). Sheppard & Vernon (2008) further showed a stronger correlation between RTT performance with fluid and crystallised intelligence than memory processing tasks with intelligence. Hence, our results and previous findings highlight the close relationship between simple motor IPS measures and cognitive ability.

Due to the MRC NSHD data set's limitations, we were unable to construct our cognitive IPS variable with more tests other than the LCT. Future research incorporating a wider range of cognitive IPS measures could provide deeper insights into these relationships, potentially reducing the observed differences between the path coefficient of motor IPS with cognitive functioning and that of cognitive IPS."

Taken together, these findings highlight that, whilst cognitive IPS is related to cognitive measures (as can be seen by the highly significant correlations between cognitive IPS and cognitive function in Supplementary Table 3), motor IPS also plays a critical role in explaining cognitive variables from test batteries.

Response to Reviewer 2's comments

1. Introduction: The rationale for this study needs improvement. It would be relevant to better explain why this study is useful, identify the research gap it aims to address in the scientific literature, and emphasize why it is important to address this gap. To better highlight the significance of the study, I suggest extending this section.

We have revised the Introduction with additional references to highlight the rationale of the current study. Specifically, we provide an overview of previous findings on the associations between IPS and cognitive variables, such as logical memory, verbal fluency, National Adult Reading Test and Letter-Numbering Sequencing (WAIS) (lines 50-55).

Following, we highlight inconsistent findings across studies. For instance, "previous research within the Lothian Birth Cohort (LBC) 1936 has demonstrated that IPS in 70-year olds, measured through IT, SRT, and CRT, serves as an indicator for intelligence, spatial and verbal abilities (Johnson & Deary, 2011)" (lines 59-62).

"However, other studies produced slightly different results. While IPS, as assessed via IT, SRT and CRT, was associated with general cognitive abilities, only SRT and CRT but not IT related to childhood intelligence (Deary et al., 2010)" (lines 62-65). We highlight that such discrepancies necessitate for "a comprehensive approach to investigate IPS and its associations with cognitive and demographic variables" (lines 65-66).

To further elaborate on this point, we present previous research on the relationship of IPS and education (lines 67-73).

"On one hand, substantial evidence links cognitive functions, including IPS, to educational attainment. For instance, the WAIS-IV standardisation study reported a mean processing speed index of 86 for individuals with less than 8 years of education, compared to 106 for those with more than 18 years (Holdnack et al., 2013). Zhang et al. (2015) also found significant associations between IPS (measured by the Digit Symbol Substitution Test) and education. On the other hand, a growth curve modelling study showed that the rate of cognitive decline, including IPS, is not associated with educational level (Ritchie et al., 2016)".

We build on this by suggesting that these discrepancies "may be due to the focus on a few selected variables for investigation, without accounting for their covariance with additional variables. For instance, Richtie et al. (2013) found that the correlation between IPS (measured via SRT, CRT and

IT) and education became non-significant after controlling for childhood IQ, measured prior to differential education at age 11. These findings suggest that while later life IPS is linked to education, childhood IQ (likely indicative of subsequent educational attainment) emerges as the primary determinant. Additionally, this raises the question of whether IPS is independently influenced by education or if observed associations are due to shared variance with intelligence" (lines 77-84).

"Together, the inconsistencies in findings underscore the necessity of not only examining pairwise correlations between variables but also considering their covariances within a statistical model. Consequently, the primary aim of this study is to employ Structural Equation Modelling to delineate the relationships between specific variables, accounting for their covariances with correlated cognitive and demographic factors. To achieve this, we set out with two main objectives: (1) To model individual differences in IPS measures at ages 60-64 (LCT, SRT/CRT) with variables known to be associated with IPS and (2) to investigate the longitudinal association of IPS with cognitive decline at ages 68-70, while controlling for health-related, demographic, and cognitive variables.

We utilise data from the Medical Research Council National Survey of Health and Development cohort, which includes measurements similar to those of the LBC 1936. By modelling separate variables to capture cognitive and motor IPS, we aim to better understand their distinct and overlapping components, addressing a gap in the literature where IPS is often treated as a single construct" (lines 106-117).

2. Cohort data: I suggest adding some information (e.g., gender, age means, sd) about the participants resulting available for the present study. Information about ethical statement and informed consent needs to be clarified.

We thank the Reviewer for their suggestion. In the Revision, we have included gender information within the 'Cohort Data' section in line 136 (1114 females out of 2124 participants) for Model 1 and in line 249 (932 females out of 1776 participants) for Model 2. Additionally, we have reported the mean ages and their standard deviations for the two main collection waves (lines 133-134), specifically for ages 60-64 (M = 63.37, SD = 1.10) and 68-70 (M = 69.50, SD = 0.23). Note that the MRC NSHD is a birth cohort study, and hence the standard deviation of age across participants is small.

We have included the information about ethics approval (lines 141-144): "Ethics approval and informed consent for the original data collection were obtained by the MRC NSHD study investigators at its inception and subsequent follow-up phases. In compliance with ethical guidelines and data access agreements, appropriate permissions were obtained from the MRC NSHD data management team at UCL to access and analyse the dataset for the purposes of this study."

3. Variables: Even if details on each measure can be found in Moulton et al. I think that may be useful add in this section more information, such as references, validation, and psychometric characteristics,

about the assessment measures. Lines 142-146, page 8: Please, specify if health indexes were self-reported or not.

In the revised manuscript, we've added a section (lines 202-219) discussing the ACE-III, NART, LCT, and reaction time tasks, emphasizing their good psychometric properties:

“While psychometric properties for the tasks used in the MRC NSHD data cohort have not been systematically evaluated, it is important to note that many of these tasks have been extensively studied in previous research, which have established their reliability and validity. The LCT exhibits high test-retest reliability (r = .93) and displays strong correlations with other assessments of IPS, such as the Trail Making test Part A and WAIS Digit Symbol test, affirming its convergent validity (Uttl & Pilkenton-Taylor, 2001).

The SRT and CRT tasks used in the current study are similar to the computerised Deary-Liewald RTTs (Deary et al., 2011). In a healthy sample aged 18-80, these tasks demonstrated high reliability, with Cronbach's alpha values of .94 for SRT and .97 for CRT on correct responses (Deary et al., 2011). Additionally, in healthy older adults, the SRT showed moderate relative variability (Inter-Class Coefficient = 0.61), while the CRT exhibited good relative variability (Inter-Class Coefficient = 0.89) (Ferreira et al., 2021).

Cognitive assessment batteries used in the MRC NSHD data cohort also show good psychometric properties. The ACE-III has high test-retest reliability for the overall score (r = .90) and individual dimensions (r = .89-.93) (Alilou et al., 2017), along with strong internal reliability (Cronbach's alpha = .88) (Noone, 2015). Moreover, the NART demonstrates high construct validity, as evidenced by its loading on general cognitive ability at 0.85 (Crawford, Stewart, et al., 1989). Regarding reliability, the NART exhibits high internal consistency (Cronbach's alpha = 0.90), as well as excellent test-retest reliability (r = 0.98) (Crawford, Parker, et al., 1989) and interrater reliability (r = 0.88) (O’Carroll, 1987). Additionally, re-standardisation against the WAIS-IV indicates robust correlations between NART scores and premorbid IQ scores (Bright et al., 2018).”

Additionally, we specified that health indexes were self-reported (line 195).

4. Pre-processing: Data preprocessing was conducted; however, why was standardization not performed?

We employed a preprocessing method of scaling the data by dividing values by 10 or 100. This was done to align the values on a similar scale, ensuring comparable variation while preserving the original (natural) metric. “This approach maintains the interpretability of the path coefficients while reducing model convergence issues due to large differences in variable variances” (lines 224-225).

In fact, SEM analyses in lavaan allow for the estimation of both unstandardised and standardised path coefficients. Alongside the unstandardised path coefficients, we report standardised path coefficients (standardised for all variables) in brackets in Figures 3 and 4. Furthermore, we provide standardised path coefficients exclusively for latent variables in the new Supplementary Tables 5 and 6 for completeness.

We also validated our preprocessing method by re-running our analyses with manually z-standardised values, yielding identical results in terms of model fit and p-values.

5. Statistical Analysis: Lines 181-188, page 10: the model 1 and the model 2 are not clear. For example, I suppose that the model 1 “Cognitive/Motor IPS = Socio-economic status + sex + intelligence + memory + smoking + BMI + exercise + CNS med. + benzodiazepines + anti-psychotic med. + anti-depressants + antiepileptic med. + anti- parkinsonian med. + neuromuscular relaxants + sedatives” should correspond with the figure at page 30. Please, could the Author(s) clarify this point? Also, for the model 2.

We have clarified our reports in model definition. In the Revision, Figure 3 shows the results from Model 1 (line 302), and Figure 4 shows the results from Model 2 (lines 315-316).

We apologise that the caption is missing which might have resulted from separate submissions of figure files and figure captions on the journal’s website. We have attached the figures, including their captions, at the end of the revised manuscript.

6. Statistical Analysis: Lines 189-196, page 10: “Model fits were determined by two measures” but three indexes were reported (i.e., CFI; RMSEA, SRMR). Furthermore, why did the author(s) choose not to report the TLI as a fit measure?

In line with the Reviewer’s suggestion (see Point 7 below), we have included the TLI and $\chi 2/df$ as additional fit measures. There are a total of five measures in the revised manuscript: CFI, TLI, RMSEA, SRMR, and $\chi 2/df$ (see lines 275 following).

7. Statistical Analysis: Lines 194-196, page 10: As the Author(s) state in the manuscript, due the highly sensitivity to the sample size $\chi 2$ is reported only for information. I suggest using the $\chi 2/df$ as a fit measure.

We have included $\chi 2/df$ values when reporting fit indexes (lines 296, 297, 301 and 313 for measurement model 1, measurement model 2, overall SEM model 1 [including measurement and structural model] and overall SEM model 2 [including measurement and structural model, respectively]. In the 'statistical analyses' section (lines 282-284), we have included a statement as to why we report $\chi 2/df$. "Nevertheless, for completeness, we report $\chi 2/df$ as a normalised measure of relative fit independent of sample size, with smaller values indicating better fit and a cut-off of 5 being a common benchmark (Hu & Bentler, 1999)."

8. Results: Lines 206-207, page 10: "(Model 1: $\chi 2(31) = 62.611$, $p < .001$; RMSEA = .022; SRMR = .014; CFI= .994; Model 2: $\chi 2$ (88) = 226.092, $p < .001$; RMSEA = .031; SRMR = .023; CFI = .975)" These results do not correspond to the results from Model 1 at page 11, lines 208-219, and Model 2 at page 11, lines 218-219. I suppose that are four different models (two for the measurements models and the other two as main analysis). It seems to be a labeling problem. Could the author(s) fix it?

The statistics the Reviewer cited above were indeed from the measurement models of the SEM Model 1 and Model 2. As in standard SEM analysis, there is one measurement model and one overall SEM model (measurement model + structural model). In the revised Results and Figures, we have clarified the names used to refer to individual models.

First, we have clarified that constructing latent variables within the measurement models was our first step (lines 293-294). We have also included the phrase "measurement model for Model 1 (or Model 2)" before reporting the fit indexes for the measurement models (lines 295-297). The measurement models from SEM Model 1 and Model 2 are illustrated in Figure 2.

Second, in lines 299-300, we have clarified that constructing the overall SEM models was the subsequent step, and that the following fit indexes pertain to the complete SEM models (including both the measurement and structural models). The complete SEM models are illustrated in Figure 3 (SEM Model 1) and Figure 4 (SEM Model 2).

9. Results: I can't see tables for main analysis, as well as caption for figure at page 28, 29, and 30. Could the author(s) add it?

We apologise that the caption is missing which might have resulted from separate submissions of figure files and figure captions on the journal’s website. We have attached the figures, including their captions, at the end of the revised manuscript with tracked changes highlighted.

In the Revision, the main SEM results are displayed in Figures 3 and 4. Additionally, we have added two tables in the Supplementary Materials, listing all coefficients and their associated values of the two overall SEM models (Supplementary Table 5 for Model 1, and Supplementary Table 6 for Model 2). We referred to those in the main manuscript (lines 316-317).

References

Alilou, M. M., Khanjani, Z., Hashemi, T., Parvaz, S., & Boulevard, B. (2017). The psychometric properties of the third version of Addenbrooke’s Cognitive Examination (ACE-III) in a sample of Iranian older adults Article Info. J Kermanshah Univ Med Sci, 21(2), 57–61.

Bright, P., Hale, E., Gooch, V. J., Myhill, T., & van der Linde, I. (2018). The National Adult Reading Test: restandardisation against the Wechsler Adult Intelligence Scale-Fourth edition. Neuropsychological Rehabilitation, 28(6), 1019–1027. https://doi.org/10.1080/09602011.2016.1231121

Crawford, J. R., Parker, D. M., Stewart, L. E., Besson, J. A. O., & De Lacey, G. (1989). Prediction of WAIS IQ with the National Adult Reading Test: Cross-validation and extension. British Journal of Clinical Psychology, 28(3), 267–273. https://doi.org/10.1111/j.2044-8260.1989.tb01376.x

Crawford, J. R., Stewart, L. E., Cochrane, R. H. B., Parker, D. M., & Besson, J. A. O. (1989). Construct validity of the National Adult Reading Test: a factor analytic study. Personality and Individual Differences, 10(5), 585–587. https://doi.org/https://doi.org/10.1016/0191-8869(89)90043-3

Deary, I. J., Der, G., & Ford, G. (2001). Reaction times and intelligence differences: A population-based cohort study. Intelligence, 29(5), 389–399. https://doi.org/https://doi.org/10.1016/S0160-2896(01)00062-9

Deary, I. J., Johnson, W., & Starr, J. M. (2010). Are processing speed tasks biomarkers of cognitive aging? Psychology and Aging, 25(1), 219–228. https://doi.org/10.1037/a0017750

Deary, I. J., Liewald, D., & Nissan, J. (2011). A free, easy-to-use, computer-based simple and four-choice reaction time programme: The Deary-Liewald reaction time task. Behavior Research Methods, 43(1), 258–268. https://doi.org/10.3758/s13428-010-0024-1

Ferreira, S., Raimundo, A., del Pozo-Cruz, J., & Marmeleira, J. (2021). Psychometric properties of a computerized and hand-reaction time tests in older adults using long-term facilities with and without mild cognitive impairment. Experimental Gerontology, 147(January). https://doi.org/10.1016/j.exger.2021.111271

Hatta, T., Yoshizaki, K., Ito, Y., Mase, M., & Kabasawa, H. (2012). Reliability and validity of the digit cancellation test, a brief screen of attention. Psychologia, 55(4), 246–256. https://doi.org/10.2117/psysoc.2012.246

Holdnack, J. A., Drozdick, L. W., Weiss, L. G., & Iverson, G. L. (2013). WAIS-IV, WMS-IV, and ACS : Advanced Clinical Interpretation (NV-1 o). Academic Press.

Hu, L. T., & Bentler, P. M. (1999). Cutoff criteria for fit indexes in covariance structure analysis: Conventional criteria versus new alternatives. Structural Equation Modeling, 6(1), 1–55. https://doi.org/10.1080/10705519909540118

Johnson, W., & Deary, I. J. (2011). Placing inspection time, reaction time, and perceptual speed in the broader context of cognitive ability: The VPR model in the Lothian Birth Cohort 1936. Intelligence, 39(5), 405–417. https://doi.org/10.1016/j.intell.2011.07.003

Noone, P. (2015). Addenbrooke's Cognitive Examination-III. Occupational Medicine, 65(5), 418–420. https://doi.org/10.1093/occmed/kqv041

O'Carroll, R. E. (1987). The inter-rater reliability of the National Adult Reading Test (NART): a pilot study. The British Journal of Clinical Psychology / the British Psychological Society, 26 (Pt 3), 229–230. https://doi.org/10.1111/j.2044-8260.1987.tb01352.x

Ritchie, S. J., Bates, T. C., Der, G., Starr, J. M., & Deary, I. J. (2013). Education is associated with higher later life IQ scores, but not with faster cognitive processing speed. Psychology and Aging, 28(2), 515–521. https://doi.org/10.1037/A0030820

Ritchie, S. J., Tucker-Drob, E. M., Cox, S. R., Corley, J., Dykiert, D., Redmond, P., Pattie, A., Taylor, A. M., Sibbett, R., Starr, J. M., & Deary, I. J. (2016). Predictors of ageing-related decline across multiple cognitive functions. Intelligence, 59, 115–126. https://doi.org/10.1016/j.intell.2016.08.007

Saito, R., Ikeda, Y., Okuzumi, H., Kobayashi, I., & Kokubun, M. (2015). Effect of Visual Field Constriction on Visual Search in Orderly Array and Random Array Cancellation Tasks. Psychology, 06(14), 1873–1878. https://doi.org/10.4236/psych.2015.614184

Sheppard, L. D., & Vernon, P. A. (2008). Intelligence and speed of information-processing: A review of 50 years of research. Personality and Individual Differences, 44(3), 535–551. https://doi.org/10.1016/J.PAID.2007.09.015

Treviño, M., Zhu, X., Lu, Y. Y., Scheuer, L. S., Passell, E., Huang, G. C., Germine, L. T., & Horowitz, T. S. (2021). How do we measure attention? Using factor analysis to establish construct validity of neuropsychological tests. Cognitive Research: Principles and Implications 2021 6:1, 6(1), 1–26. https://doi.org/10.1186/S41235-021-00313-1

Uttl, B., & Pilkenton-Taylor, C. (2001). Letter cancellation performance across the adult life span. Clinical Neuropsychologist, 15(4), 521–530. https://doi.org/10.1076/clin.15.4.521.1881

Zhang, M., Gale, S. D., Erickson, L. D., Brown, B. L., Woody, P., & Hedges, D. W. (2015). Cognitive function in older adults according to current socioeconomic status. Http://Dx.Doi.Org/10.1080/13825585.2014.997663, 22(5), 534–543. https://doi.org/10.1080/13825585.2014.997663

VERSION 2 – REVIEW

**REVIEWER NAME**	Roivainen, Eka
**REVIEWER AFFILIATION**	Oulu University Hospital
**REVIEWER CONFLICT OF INTEREST**	None.
**DATE REVIEW RETURNED**	26-Jun-2024

**GENERAL COMMENTS**	This study reports interesting observations involving IPS and other cognitive factors measured at a younger age and their association with cognitive performance at older age.<br><br>I find the response to my previous comments satisfactory.<br><br>Obviously, the large sample of participants, the longitudinal approach, and the statistical analysis are the strong points of this study.<br><br>The assortment of cognitive tests used looks as the weak point of this study. A miscellany of tests were used, some of them popular and with proven validity, others with weaker psychometric credentials.<br><br>However, I think the results do convincingly enough support the authors conclusion of distinct types of IPS that may relate to different cognitive and SES factors in diverse ways.<br><br>I recommend the publication of this reviewed manuscript

**REVIEWER NAME**	Ricciardi, Elisabetta
**REVIEWER AFFILIATION**	University of Bari, Educational Sciences, Psychology and Communication
**REVIEWER CONFLICT OF INTEREST**	None.
**DATE REVIEW RETURNED**	12-Jul-2024

**GENERAL COMMENTS**	I have reviewed the revised manuscript and believe it has improved. Thank you for addressing the feedback provided.

VERSION 2 – AUTHOR RESPONSE

Response to Reviewer 1’s comments

1. I find the response to my previous comments satisfactory. […] I recommend the publication of this reviewed manuscript.

We thank the Reviewer for their comments and recommendation of publication.

Response to Reviewer 2’s comments

1. I have reviewed the revised manuscript and believe it has improved. Thank you for addressing the feedback provided.

We thank the Reviewer for their confirmation that the revised manuscript has addressed their previous feedback.
